# An Empirical Exploration of Volunteer Management Theory and Practice: Considerations for Sport Events in a “Post-COVID-19” World

**DOI:** 10.3389/fspor.2022.689209

**Published:** 2022-03-11

**Authors:** Gareth Power, Olesya Nedvetskaya

**Affiliations:** Cardiff School of Sport and Health Sciences, Cardiff Metropolitan University, Cardiff, United Kingdom

**Keywords:** volunteering, events, management, sport, COVID-19, pandemic, volunteer management, large sport events

## Abstract

The existing literature highlights some universal principles that are widely accepted as a good volunteer management practice, e. g., volunteer appreciation and recognition, provision of meaningful roles, effective communication, and the importance of relational connection, just to name a few. Yet, it can be argued that a gap exists in the relationship between volunteer management theory and practice in the context of large sport events. For example, published evidence shows that volunteer programs often lack effective planning and management to achieve successful program results. On one hand, this can be attributable to limited knowledge about volunteers' characteristics and motivations, their lived experiences, the processes of volunteering, and the actual volunteer management practices. On the other hand, a lack of the right mechanisms (e.g., political will, financial, and managerial resources) in place before and throughout the event lifespan intensifies this disconnect. The aim of this research, therefore, was to critically examine the reasons behind this theory-practice divide in volunteer–volunteer management relationship and its potential impact on volunteer experiences and volunteer program outcomes, particularly in the context of the COVID-19 pandemic and its implications for sport event organizers and volunteer managers in the UK. A mixed methods approach was adopted for this study: a survey conducted with volunteers (*n* = 101) combined with a series of interviews with volunteers (*n* = 8) and volunteer managers (*n* = 6). The study identified some potential challenges facing volunteer programs associated with large sport events post-pandemic, particularly in relation to volunteer recruitment, volunteer management, and safety concerns affecting volunteer confidence to re-engage in volunteering. These challenges carry with them certain resource implications that event organizers need to consider to effectively run volunteer programs and support volunteers in engagement and re-engagement following the pandemic, as well as to harness opportunities the pandemic has potentially created to successfully re-emerge from the shadow of COVID-19. The study provides specific recommendations to inform event planning and delivery to enhance volunteer experiences and, ultimately, outcomes of volunteer programs associated with large sport events.

## Introduction

With an estimated equivalent value of £22 billion, volunteering contributes significantly to society in the UK (Office of National Statistics, 2017). Furthermore, within the UK sport sector volunteers contribute an annual equivalent value of more than £1.5 billion (Coleman, 2002). The volunteer workforce is arguably the most valuable asset possessed by the sector and is acknowledged as the indispensable resource for the operational and economic success of sport events (Catano et al., 2001; Surujlal and Dhurup, 2008; Nichols, 2014). Miles and Shipway (2020) acknowledged that the impact of the COVID-19 pandemic on the sport sector remains largely unknown. However, any impact upon the sector's volunteer workforce should be considered within the context of decline among those who volunteer in formal environments in the UK (Cuskelly et al., 2006a; Sport Recreation Alliance, 2018; Department of Media Culture Sport, 2019). These decreasing levels of engagement in formal volunteerism, along with the potential impact of COVID-19 present serious challenges to a sector already under a financial strain, and a threat to the successful delivery of sport events (Hiltrop, 1999; Cuskelly et al., 2006a; Studer and von Shnurbein, 2013; Sheptak and Menaker, 2020).

Effective volunteer management, therefore, has a crucial role to play in the successful re-emergence of the sport events industry from the pandemic. It is widely acknowledged that effective management of volunteers can influence positively volunteer experiences, performance, and likelihood of continued engagement (Allen and Bartle, 2013; Nedvetskaya and Girginov, 2017). This, in turn, can enhance the quality of sport events through the maintenance of a consistent workforce, increased efficiency, and enhanced spectator/participant experience (Taylor et al., 2008; Hallmann et al., 2018). Hence, a better understanding of volunteer attraction and management “on the ground”, and the resources required to do so effectively would benefit volunteers, event organizers, and the sector alike. Existing research broadly agrees on key themes that influence volunteer motivation and retention and the effective implementation of good volunteer management practices, i.e., meaningful volunteering roles, quality of experiences, levels of appreciation, effective relational connection, and clear communication (Allen and Bartle, 2013; Dunn et al., 2016; Nedvetskaya and Girginov, 2017). Yet, it appears that in practice volunteer recruitment and drop-off remain among the main concerns for sport event organizers with unsatisfactory volunteer management highlighted as a key contributor to volunteer dissatisfaction (Studer and von Shnurbein, 2013; Nedvetskaya and Girginov, 2017; Nichols et al., 2019). This seems to be exacerbated by the COVID-19 pandemic which put a halt to many sport events and volunteer activity, with the immediate and longer-term impact on the sport sector and its workforce currently unknown (Sheptak and Menaker, 2020).

This study aimed to address research questions related to (a) the theory-practice divide in volunteer–volunteer management relationship in the UK context of large sport events, and (b) the implications of the COVID-19 pandemic for volunteer programs related to recruitment, training, supervision, and recognition of volunteers. Firstly, the focus was on the broader organizational context of volunteer management and the exploration of barriers to the effective implementation of known good practices. The intention was to identify the extent to which a disconnect exists between theory and practice of volunteer management, the reasons behind it, and provide recommendations to inform event planning and delivery to enhance volunteer experiences and volunteer program outcomes. Secondly, close consideration was given to the impact of the COVID-19 pandemic on sport event volunteers, their likelihood to engage or re-engage in event volunteering post-pandemic, and the implications for volunteer managers and event organizers as the industry continues to re-emerge from this crisis.

## Literature Review

### Sport Event Volunteering

Volunteering in the context of sport events has been the subject of extensive research literature (Hallmann and Harms, 2012; Gallarza et al., 2013). Much of this research revolves around the types of people who volunteer and their motivations (Kirstiansen et al., 2015; Schlesinger and Nagel, 2018). Despite this strong focus on the socio-psychological dimension of volunteering, there have also been numerous studies identifying the theory of good practice from a volunteer management perspective (Musick and Wilson, 2008). Kim et al. (2018) asserted that a substantial amount of this research can be categorized into two broad areas—volunteer characteristics and volunteer management.

### Volunteer Characteristics

It is widely agreed that the volunteer demographic is diverse, comprised of people from different socio-economic backgrounds, cultures, nationalities, genders, ages, ethnicities, and personalities (Alexander et al., 2015). As such, volunteers present a diversity of motives, expectations, behaviors, and needs (Alexander et al., 2015). According to Cuskelly et al. (2006a), volunteer motivations are multifaceted in nature and can be located on a continuum, positioning altruistic and egoistic motivations at opposing ends. Indeed, Yeung (2004) highlighted a variety of differing volunteer motivations including altruistic reasons, the desire for social contact, the pursuit of personal interests, and fulfillment of emotional needs. Additionally, other influences such as family tradition, group identification, and the desire to utilize personal skills to contribute positively to society are also key factors affecting volunteer motivation (Bang and Ross, 2009). Kirstiansen et al. (2015) pointed to the challenge this presents to sport volunteer managers as they attempt to balance various needs and motivations of different volunteers. The more effectively this is achieved, the better the outcome for both the volunteers and the sport event organization (Taylor et al., 2006; Allen and Bartle, 2013; Nedvetskaya et al., 2015).

Cuskelly et al. (2006b) suggested that the adoption of a traditional Human Resource Management (HRM) approach when dealing with volunteers in the sport and recreation sector has its limitations as the effectiveness is dependent on varying factors, including volunteer motivations. As a result, an alternative approach is endorsed that considers volunteer motivation and the situational context of the volunteer environment. This contrasts with a paid workforce motivated by extrinsic factors and contractual obligations (Cuskelly et al., 2006b). This argument adds to the findings of Shin and Kleiner (2003) who proposed that, unlike paid employees, volunteers are more motivated by intrinsic reward and, therefore, any HRM approach should be reflective of this. As a result, a volunteer-centered approach focusing on volunteer motivations could positively impact volunteer satisfaction and, ultimately, retention. This underlines the importance of effective management of volunteer programs associated with sport events, particularly where long-term volunteering is concerned (Cuskelly et al., 2006b; Kirstiansen et al., 2015).

### Volunteer Management

Event managers with responsibility for volunteers play a key role in influencing the experience, expectations, and motivation of volunteers (Allen and Bartle, 2013). Volunteer management roles are concerned with the recruitment, orientation, deployment, and retention of volunteers (Studer and von Shnurbein, 2013). The volunteer recruitment process, along with the volunteer management before, during, and post-events are key factors in successful event delivery and the creation of positive volunteer experiences (Taylor et al., 2006). Volunteer orientation and training are particularly considered key components of volunteer programs (Taylor et al., 2008) that can help reduce stress, make volunteers feel welcome, and enhance their retention (Australian Sports Commission, 2000, cited in Taylor et al., 2008).

According to Hoye et al. (2018), good communication, respect, clear expectations, and flexibility are valued elements of effective leadership. This can increase engagement and commitment from a workforce (Hoye et al., 2018). As a result, the demonstration of such leadership principles should be evident in the practice of volunteer managers to effectively engage volunteers. Additionally, the recognition of volunteers is acknowledged as an important way to maintain their engagement (Cox, 2002). Perks such as volunteer uniforms, certificates, and awards evenings are identified as good practices (Cox, 2002; Phillips and Phillips, 2010). Moreover, one of the most valuable things volunteer managers can do is take time to express gratitude and show interest in volunteers on a personal level (Phillips and Phillips, 2010). This investment may have no financial bearing but does require sufficient human capacity to invest in volunteers in this way, which may only reinforce a positive volunteer-manager relationship claimed to be a key component of strong volunteer engagement (Kim et al., 2018). Importantly, according to Taylor et al. (2006), this depends on how well volunteer management processes are designed and implemented, as they require the corresponding human resources to be effective (Cuskelly et al., 2006a). Further research is endorsed to enhance understanding of the relationship between volunteer motivation, behavior and volunteer management practices (Cuskelly et al., 2006b; Allen and Shaw, 2009; Allen and Bartle, 2013).

### COVID-19 Pandemic, Sport Sector, and Volunteering

While the long-term impact of the COVID-19 pandemic on the sport sector is currently unknown, it is evident that it significantly disrupted the global society and the sport industry alike (Sheptak and Menaker, 2020). The World Health Organization, national and local Governments, sport governing bodies across the globe all responded to COVID-19 with various measures and restrictions, including a suspension of mass gatherings (e.g., to limit the spread of the virus and allow for more rapid vaccination programs to take place), which resulted in either cancellation or postponement of many sport events, including UEFA EURO 2020 and the Tokyo 2020 Olympics (Olympic Games, 2012; Miles and Shipway, 2020; Tokyo, 2020; Wackerhage et al., 2020). These decisions have not only negatively affected sport fans and elite athletes, but also the workforce that helps make these events happen, especially considering the positive role of sport events in providing identity, connection, and satisfaction (Sheptak and Menaker, 2020).

Indeed, work, whether through employment or volunteering, is known to increase a person's self-esteem, sense of fulfillment, and feeling of belonging. It is widely acknowledged that volunteering brings benefits that transcend tangible, extrinsic rewards (Cuskelly et al., 2006b; Allen and Bartle, 2013). The suspension of many sport events has presumably impacted volunteers' wellbeing, which may have implications for event managers as the sector re-emerges from the pandemic. This highlights the need for closer consideration of the connection between the pandemic and socio-psychosocial implications for volunteers, their wellbeing, and, ultimately, the quality and effectiveness of the work they perform—key components of successful volunteer programs associated with sport events. This is in line with the call for further studies into the response and resilience of sport events to unexpected challenges to solidify the research base and help the sector navigate its way out of this crisis (Miles and Shipway, 2020; Sheptak and Menaker, 2020).

### Research Focus

Despite considerable studies published on event volunteering, it remains an emerging field of research (Hallmann and Harms, 2012). The present study aimed at adding to the limited literature on the relationship between theory and practice of volunteer management and the contextual factors that influence it. While many studies focus on the motives, dispositions, and characteristics of volunteers (Studer and von Shnurbein, 2013), there is an insufficient focus on wider organizational characteristics and contexts within which volunteers function (Cuskelly et al., 2006a; Musick and Wilson, 2008; Roza et al., 2017; Schlesinger and Nagel, 2018). While existing research is concerned with individual sport events (Hallmann et al., 2018), the current study considered multiple event contexts, gleaning from a broader range of experiences and environments to provide an alternative angle of research. Despite volunteers being acknowledged as a crucial element of the workforce to help deliver large sport events (Cuskelly et al., 2006a; Nichols, 2014), increasing concerns exist about volunteer capacity for sport events, and especially so in the context of the COVID-19 pandemic (Miles and Shipway, 2020; Sheptak and Menaker, 2020), which is an unprecedented phenomenon that provides a unique situation in its own right. Thus, close examination of the volunteer–volunteer management relationship helps identify critical factors affecting the disconnect between theory and practice and what this means for sport events and their volunteer programs that are challenged by the COVID-19 pandemic yet strive to re-emerge from the crisis.

## Methods and Sampling

A case-study research approach was adopted for this research to allow for an empirical examination of volunteer experiences and management within a sport event context. The goal was to gather valuable insights based on personal views, opinions, and experiences to help understand the behavior of volunteers and volunteer managers and the environmental and contextual factors that affect them. Due to the social dimensions and the organizational context involved in this study, an empirical, mixed methods approach was adopted consisting of predominantly qualitative data (Skinner et al., 2015) yet complemented by a quantitative approach to have a richer and stronger array of evidence.

Quantitative data was captured *via* a standardized Qualtrics-based online survey consisting of two sections. The first section focused on volunteer motivations, experiences, and individual perceptions of volunteer management. The second section focused on the impact of the COVID-19 pandemic on volunteer activity, health and wellbeing, and the factors affecting volunteers' likelihood of re-engaging in event volunteering post-pandemic. The survey comprised mostly closed questions, with some selected open-ended questions used to gather wider insights about volunteer experiences of management practices (e.g., communication, volunteer training, supervision, and recognition). A draft survey was compiled, peer-reviewed, and validated allowing for modification before circulation to help ensure effective data collection. The survey was circulated to 962 volunteers registered with Cardiff Council's “Volunteer Sports Bureau”. This bureau is used to promote sport-related volunteer opportunities in Cardiff (UK), including major sport events.

The survey data was captured between the 16th−30th of December 2020, when the UK was under high levels of social restrictions due to COVID-19. The survey received 127 responses, yielding 101 usable results (13.2% response rate). Importantly, while 100% of survey respondents had first-hand experience of event volunteering, a proportion of the volunteers registered with the “Volunteer Sports Bureau” would not have been “event volunteers” but rather volunteers in non-event environments (e.g., local sports clubs). These volunteers were therefore ineligible to take part in the survey. It was not feasible to differentiate between the “type” of volunteer the survey was circulated to using the Bureau, which would have contributed to the relatively low response rate, combined with the survey overlapping the December holiday period in the UK. Yet, the survey design allowed for attracting only sport event sector volunteers, and therefore, all survey respondents had previously volunteered at least once at a sport event with 49.5% of them having volunteered at five events or more. A further 5.9% had volunteered at four events, 14.9% at three events, and 12.9% at two events. As a result, a range of volunteer experience levels was incorporated into the study, helping reduce the risk of response bias among the respondents. The survey participants consisted of 60 females (59.40%), 40 males (39.60%), and one non-disclosure (0.99%). Respondents were all aged 18+. Further volunteer demographics are shown in Table A1. 43.6% of survey respondents were in full-time employment, with the next largest demographic being people who were retired (22.8%). Students made up 7.9% of the population sample. Given the relatively small sample size and low-level complexity of the survey, the quantitative analysis for this study was conducted using descriptive analysis performed *via* MS Excel spreadsheet software.

Semi-structured interviews with volunteers and volunteer managers (*n* = 14) enabled capturing deeper insights by digging “beneath the surface” of the more generalized survey responses to understand various perspectives as well as contextual and organizational challenges. This helped better understand the practice of volunteer management and how it was perceived by volunteers, the impact of COVID-19 on both volunteers and managers, particularly around new management practices that had to be created and implemented to respond to this threat yet help the sector survive. All interviews were conducted between the 13th of January and 3rd of February 2021 during the height of the COVID-19 pandemic in the UK. To minimize the risk of the virus transmission, all interviews were conducted *via* a video link using Microsoft Teams. Interview questions were divided into two sections, with the first section exploring volunteer motivations, their volunteering experiences, and perceptions related to volunteer management during sport events. The second section focused on how the pandemic was affecting volunteers in terms of their levels of volunteer activity, health, and wellbeing. This section also considered factors affecting the confidence and motivation of volunteers to return to event volunteering post-pandemic and explored practical considerations for volunteer managers that may influence future volunteer recruitment and management approaches. All volunteer and volunteer manager interviews followed similar themes and structures to enable comparisons among various perspectives. The key difference was in volunteer interview questions being focused on the impact of the pandemic on volunteers' health and wellbeing, whereas volunteer manager questions being focused on the operational challenges and the organizational context within which managers operated. Interview questions were peer-reviewed and validated accordingly before the start of the interview process. The use of scripts to introduce the subject helped provide consistency across different interviews, ensuring important points were covered and that assurance of confidentiality was provided to encourage openness and honesty in participant answers (Gratton and Jones, 2010).

A purposive approach was adopted in selecting the interview sample to help answer the proposed research questions. Using the Volunteer Sports Bureau, eight volunteers (*n* = 8) were identified based on their range of experiences and the appropriateness of the opportunities they had been involved in. All volunteer interview participants were aged 25+ with an average age of 51. Six female and two male volunteers were interviewed. Volunteer experiences included a range of different opportunities provided by the city of Cardiff that attracts internationally and nationally recognized sport events, e.g., London 2012 Olympic and Paralympic Games, UEFA Champions League Final 2017, Rugby World Cup 2015, Velothon Wales, Gemau Cymru, Cardiff Half Marathon, Volvo Ocean Race 2017, Homeless World Cup 2019, European Hockey Championships, Super League Netball, and Ashes Cricket. Additionally, the network of contacts possessed by the authors within the industry allowed for accessing six professionals (*n* = 6) from the sport sector. These individuals (four females and two males) were selected based on their rich volunteer management experience and the specialist knowledge they possess within the context of national and international sport events. These events were predominantly Cardiff-based, although volunteer managers also had experienced events across the UK and Europe. Interviews in total equated to 11:08 hours of recorded conversations that were transcribed verbatim, resulting in 162 pages (91,901 transcribed words) of rich data for analysis, which produced enough corroborating evidence to suggest that saturation was reached within this number of participants. NVIVO 12 software assisted in qualitative data management and analysis. Data was coded into key themes including “volunteer experience,” “what volunteers value,” “motivators,” “de-motivators,” “factors affecting volunteer management practice,” “return to volunteering,” “health and wellbeing,” “COVID-19 impact,” “pandemic considerations,” and “management implications.” Thematic analysis was utilized to generate codes, review, and cluster themes, and translate them into a narrative account (Braun and Clarke, 2012). Both deductive and inductive approaches were in constant interplay to produce this research. The overarching themes and the analysis itself were theory-driven whereas coding originated from the raw material and was based on participants' personal stories, reflecting their language (DeWalt and DeWalt, 2011). This formed an initial (albeit evolving) conceptual framework to support the research process and help make sense of the data. This approach enabled the drawing of initial conclusions that were verified throughout the remaining research process (Braun and Clarke, 2012).

This study was reviewed and approved by the Ethics Committee, in line with the Cardiff Metropolitan University Ethics Framework. All survey participants received written information concerning the study before providing consent, and subsequent completion of the survey. Survey responses were all anonymous. Interview participants received both written and verbal information concerning the study and provided written consent before their participation. All participants were informed of their right to withdraw from the study at any time.

## Results and Discussion

This section is split into two parts to address posed research questions related to the volunteer–volunteer management relationship associated with large sport events and implications of the COVID-19 pandemic.

### Volunteer–Volunteer Management Relationship

The results of this study showed that most survey respondents (80.2%) were generally satisfied with their volunteer experience at large sport events in the past, reporting on their experience being “very positive”. The overall rating on event experiences had a mean score of 4.17 out of 5, with a standard deviation (SD) of 0.94. These trends correlated with results from the volunteer interviews, with respondents reporting generally positive experiences yet with occasional negative comments. The extent to which this experience was either positive or negative depended on several key factors that had to do with personal interaction and communication, overall event organization and the management approach pre, during and post-event—the factors that find support in the literature (Cuskelly et al., 2006a; Kirstiansen et al., 2015).

This study evidenced that a volunteer's interaction with other people had the most significant effect on surveyed volunteers and their experience with a mean of 4.57 out of 5 (SD = 0.67)—whether that was interaction with the public, participants, volunteer managers, or fellow volunteers. This finding was reinforced by the volunteer interviews with one volunteer stating “…*one of the biggest things for me is the connection with the people you're helping, and the connection with the team around you*” (Volunteer D). In support of Taylor et al. (2006) assertions, it also appeared that the recruitment phase of volunteer programs along with pre-event communication were key factors in shaping volunteer experiences. Where these elements were deemed poor, volunteer experience was negatively impacted: “*[The organisation] were very slow …the roles weren't allocated until really close to the [event]. People didn't want to book [travel] until they knew what their role was …[my friend] decided not to go because she didn't think the organisation was very good*” (Volunteer H). This finding reinforces the need for maintaining close communication with volunteers at the key stages of the volunteer program. Equally, while post-event follow-up seemed to have a little direct bearing on volunteer experiences, this did form part of volunteer expectations with one volunteer remarking: “*You always get an email or something to say thank you for participating, sometimes you get a gift… and maybe a follow-up email to say if there are other similar volunteer roles in the future*” (Volunteer H). Given that lack of recognition and volunteer inconvenience are limiting factors to volunteer retention (Doherty, 2009), it can be inferred that post-event communication would be an advisable practice for volunteer managers showing appreciation of volunteer work and signposting volunteers to future opportunities, thereby maintaining their loyalty and continued commitment.

This study results further suggested that a proper match between volunteer motivations, experiences and role allocation is critical in both volunteer satisfaction and performance which, ultimately, has implications for volunteer management and program outcomes. Interview participants, for example, generally agreed that being involved and supporting an event was more important than the role given, which was echoed by survey respondents. Yet, further probing revealed that the ability to feel they make a useful contribution to the event was important: “*[The reason I loved the volunteer experience was that] I felt I was doing something worthwhile, I felt it was something I was doing to support people*” (Volunteer E). These findings align with existing research, suggesting a general willingness among volunteers to fulfill whatever role is necessary. But the provision of stimulating roles can increase volunteer engagement, motivation, and satisfaction (Allen and Shaw, 2009; Allen and Bartle, 2013), whereas the absence of such fulfillment was connected to more negative volunteer experiences. Interestingly, role allocation was generally considered by volunteer managers as one of the key factors in the successful delivery of effective volunteer programs: “*When [role allocation] is done well, the execution of the event runs smoothly, and the volunteer experience is higher quality. When it's done poorly, it can lead to a poor experience and the needs of the event aren't always met*” (Volunteer Manager B). This was attributed to the perception that volunteers would be more motivated in fulfilling roles, and that the event delivery would run smoother where volunteer skills, abilities, and personalities were effectively aligned to the jobs assigned. This thinking was supported in the literature, where role allocation related to volunteer characteristics, skills and motives is endorsed for the mutual benefit of both a volunteer and an event (Khoo and Engelhorn, 2011; Allen and Bartle, 2013). However, it is equally acknowledged that there are challenges associated with achieving this aspiration, including time and human resource factors (Allen and Bartle, 2013; Nedvetskaya et al., 2015).

**Figure A1:**
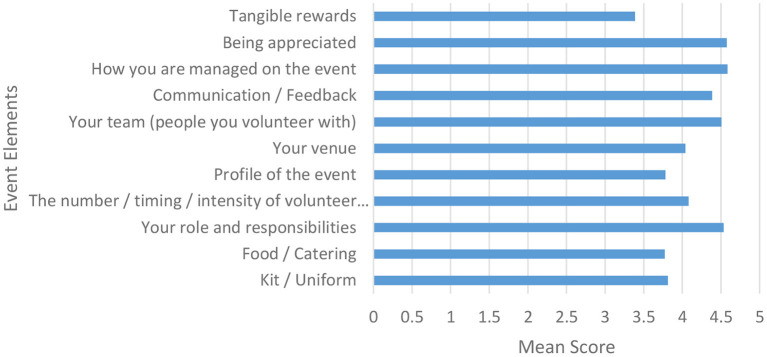
Importance of volunteer experience in sport events.

In connection to having appropriate lines of communication and being properly “matched”, this study revealed the importance of “feeling valued” for most respondents, with 93.8% of surveyed volunteers supporting this affirmation. This finding was backed by the volunteer interviewees expressing similar positive feelings: “*I didn't feel like I was somebody who's just there to make up the numbers*” (Volunteer C); and: “*[Volunteer managers] are always really thankful for you being there and you always feel wanted*” (Volunteer H). In support of the findings by Phillips and Phillips (2010), it was the people-management approach taken by volunteer managers that had the biggest impact on positive volunteer experiences, along with the allocation of meaningful roles (see Figure A1). Tangible rewards such as the provision of a quality uniform and mementoes were generally less of a determinant for feeling valued. However, these elements were more highly acknowledged by the younger volunteer demographic, whereas older volunteers appeared to have a greater appreciation of being able to make a positive contribution to events. Elements of volunteer reward identified by Cox (2002) and Phillips and Phillips (2010), such as the provision of uniforms, certificates, and “thank you” events, appeared to be common practice. This was identified by both volunteers and volunteer managers, although post-event recognition initiatives were more common among larger-scale events. This was attributed to the additional resources and planning required to host such functions.

On the other hand, volunteers expressed strong sentiments against feeling un-needed, under-appreciated or under-utilized, with one volunteer describing not being effectively employed as “*a complete waste of time*” (Volunteer G), with another stating: “*It's like… I've done a lot of volunteering over the years, and this isn't the best use of my skills …you think why did I bother*” (Volunteer E). The positive impact that intrinsic satisfaction can have on volunteer motivation is well documented in the literature (Cuskelly et al., 2006a; Allen and Bartle, 2013), and an awareness of this connection was evident among interviewed volunteer managers. However, the divergent priorities and organizational expectations they faced appeared to result in event delivery objectives becoming their primary concern, which is in line with the core purpose of event volunteer programs to aid in the successful delivery of an event. Yet, volunteer managers are seriously challenged with the need to balance the delivery of events and the interests of volunteers and their greater autonomy (Costa et al., 2006; Fairley et al., 2007; Nedvetskaya and Girginov, 2017). This would appear to be a downside of a more traditional HRM approach. Cuskelly et al. (2006b) warned of the dangers posed by regarding volunteers as a replaceable resource when preoccupation with delivering “outcomes” is legitimized at the expense of the fulfillment of volunteer needs and expectations. This in turn could have a detrimental effect on the event's success, as failure to sufficiently manage volunteer expectations can negatively impact volunteer performance and retention (Allen and Shaw, 2009; Allen and Bartle, 2013; Nichols et al., 2019).

One such example was the desire of volunteer managers to mitigate against volunteer attrition and ensure sufficient volunteer numbers to meet event requirements through deliberate over-recruitment: “*There is always a dropout, so you always have to oversubscribe your volunteer base to allow for those dropouts*” (Volunteer Manager A). While this may seem a logical practice to combat a recognized challenge of sport event volunteer attrition (Allen and Bartle, 2013), it can negatively impact volunteer experiences. As mentioned, feeling useful is a contributing factor to positive volunteer experiences. Conversely, the absence of such feelings of worth would, therefore, be a de-motivator. As one volunteer articulated: “*Sometimes there are too many of you, and you think do I really need to be here?*” (Volunteer H), with another adding: “*They always ask for too many volunteers …standing around not doing anything, that's a complete waste of time*—*then you lose people*” (Volunteer G). Based on this research evidence, it could be suggested that a careful balance should be found by volunteer managers in trying to manage volunteer attrition rates, where event needs are met while a surplus of volunteers lacking meaningful roles is avoided. Otherwise, the imbalance could negatively affect the satisfaction and associated continued engagement of volunteers motivated by a desire to contribute meaningfully, thereby adding to the volunteer drop-off problem that over-recruitment was intended to mitigate (Finkelstein, 2008; Bang and Ross, 2009; Allen and Bartle, 2013). It is regarded as a misconception that volunteers contribute to events by simply being there (Millette and Gagné, 2008). Furthermore, occupying a surplus of volunteers was identified as a drain on the time and resources of volunteer managers: “*In some instances, we probably had too many [volunteers] and making sure that they felt valued and had enough to do was one of the biggest challenges*” (Volunteer Manager F). Such observations find support in the literature where Farmer and Fedor (1999) highlight the potential for volunteers to become burdens to event organizers. This further points to the need for event organizers to re-think their approaches to volunteer recruitment and the practice of volunteer over-recruitment and its management. Indeed, volunteer recruitment, communication, and the role allocation process are acknowledged among the biggest challenges in volunteer management (Studer and von Shnurbein, 2013; Nichols et al., 2019).

To appropriately address the identified challenges, this study argues that volunteer programs should be sufficiently resourced both financially and staff wise, meaning having enough volunteer managers working with volunteers daily throughout the even life cycle—from planning to delivery and beyond. Albeit there was general agreement that volunteer programs were sufficiently resourced, 35.6% of survey respondents felt that provision of expenses or support with travel was lacking, pointing toward the importance of providing financial help to cover basic costs associated with volunteering. Albeit in demand, this practice is not widely adopted by event organizers, which highly depends on the event status and context. High costs along with insufficient communication and lack of effective management are acknowledged among the main factors that lead to volunteer dissatisfaction and dropouts (Nedvetskaya and Girginov, 2017).

Additionally, an issue with staff availability on shifts was raised as another important element contributing to the level of satisfaction/dissatisfaction. When asked what could improve their experiences, volunteers identified enhanced supervision through lower volunteer–supervisor ratios as an area to be further developed, with one volunteer stating they would “*perform better with more interaction with a team leader*” (Volunteer F). This finding is linked to the need for personal connection identified earlier. Importantly, the desire for greater supervision did not negate the need for volunteers to maintain a degree of autonomy, with one volunteer objecting to being treated as “*someone who didn't have a mind*”, saying: “*you need to have the acknowledgment that you are a person with a brain, that you have something to offer*” (Volunteer C). This further highlights the importance for volunteer managers to have the capacity to invest in getting to know and understand volunteers and developing a two-way relationship with them. Furthermore, volunteer managers accepted that staffing resources were not always adequate, with one describing this as “*definitely an area that could be worked …having a bit more resource at that level would really help*” (Volunteer Manager A). This seemed to become particularly evident when something unexpected occurred during an event requiring additional staff attention, as expressed by one interviewee: “*Events are typically resourced well enough from the human resource perspective until something changes or goes wrong! …you only really know [it] when something goes wrong*” (Volunteer Manager B). These findings suggest that there is limited additional capacity built into staffing resources for volunteer supervision to deal with unexpected occurrences.

Similarly, the pre-event phase of volunteer programs should be adequately supported in terms of human resources (Kim et al., 2018). However, four out of the six interviewed volunteer managers reported holding other competing organizational responsibilities outside of the volunteer program during the build-up to events. This appeared to heighten the pressures experienced by volunteer managers and inhibit their ability to effectively administer the volunteer programs, with one volunteer manager stating “*[I had experienced] challenges around lack of support from my organisation in terms of realising how much work I was actually doing—it was not necessarily difficult work responding to hundreds of volunteer emails, but it is time-consuming! I had other responsibilities as well and I think the day-to-day support when it comes close to the events is very important for volunteer managers*” (Volunteer Manager A). This feeling was echoed by another volunteer manager charged with divergent responsibilities that impaired their ability to deliver the level of volunteer management practice they would have liked due to performing a volunteer management role on top of their main role: “*[This was] additional responsibility which was a challenge to devote the right level of time and energy to…it was very difficult to try and effectively manage the responsibilities attached to the management of volunteers and my main role*” (Volunteer Manager F). These findings could account for a potential disconnect between volunteer managers' *knowledge* about best practice and their *behavior* in practice, suggesting that investment in additional human resources may be required by event organizers at every stage of volunteer programs to be effective. Cuskelly et al. (2006b) posited that effective HRM practices improve volunteer retention subject to appropriate resources required to effectively implement these practices. The financial investment to increase resource allocation in support of volunteer managers could, therefore, better enable them to invest in pre-event communication, role allocation, orientation, training, management, and post-event communication. This in turn could result in enhanced volunteer experience before, during and after events, thus having a positive impact on volunteer satisfaction, performance, and retention (Taylor et al., 2006; Allen and Bartle, 2013).

### Post-pandemic Implications of COVID-19

The emergence of the sport event industry from the pandemic is expected to present new challenges that will need to be navigated carefully to ensure both the welfare of volunteers and the successful delivery of sport events (Sheptak and Menaker, 2020; Wackerhage et al., 2020). None of the volunteers taking part in the study reported doing any event volunteering during the pandemic. While this was largely due to a lack of opportunity and awareness, it was also down to the fear of contracting the COVID-19 virus and the risk this may pose for others—an area that appeared to be people's primary concern about engaging in volunteer activity.

The COVID-19 pandemic has had a substantial impact on the overall wellbeing of volunteers. Almost half of the survey respondents (48.5%) reported a decrease in physical activity levels. This may be unsurprising considering how the pandemic has restricted the use of recreational facilities (e.g., gyms), swimming pools, and play areas, along with increasing numbers working from home and shopping online (Shahidi et al., 2020). Conversely, almost a quarter of volunteers (24.7%) reported doing more physical activity. This could be due to the rise in exercise taking place in homes and outdoor spaces (Smith, 2020). There were similar results concerning the general health of volunteers during the pandemic, where over half of respondents (55.4%) reported poorer health during this period. Such a correlation between decreased physical activity and health is well known and would certainly be supported by the literature (Dwyer et al., 2020). It has already been widely acknowledged that the pandemic has had a negative impact on global mental health (Torales et al., 2020). This study specifically considered the impact of having the opportunity to volunteer at sport events taken away. Almost half of the respondents (47.4%) reported some form of negative mental impact. Of these, 6.7% reported a severe impact, all of whom were male. None of the interviewed volunteers explicitly admitted that being unable to volunteer had negatively impacted their mental health. This outcome could have been influenced by the stigma associated with the subject of mental health and a potential reluctance among interviewees to discuss openly in the context of a recorded interview (Jones, 2015; MIND., 2017; UK Government, 2019), which may explain the discrepancy between the survey and interview data.

A sense of loss of the social element associated with the event volunteering experience was captured across both the survey and interview data. Interviewees, in particular, acknowledged missing social interaction and anticipation that involvement in events provided: “*I do miss the excitement of doing the events, I look forward to doing them*” (Volunteer C), and “*I definitely haven't got something to look forward to without the events… Events [are] something you look forward to doing …Because the events have stopped, it's that new experience, that is something different, that once-in-a-lifetime opportunity that has been taken away from you*” (Volunteer D). It can be suggested that these reported feelings were magnified by the current context where people are experiencing restrictions due to the pandemic. Based on these findings, it can be inferred that volunteers would be eager to return to volunteering post-pandemic or whenever the epidemiological situation would significantly improve to allow for re-opened doors. Indeed, an increased desire for social interaction is a recognized motivation for event volunteering (Lee et al., 2016), which could, therefore, prompt fresh engagement in volunteerism. Yet, given the context, this appears to be subject to such volunteer characteristics as age and gender as well as COVID-19 related environmental, administrative, and managerial factors.

**Figure A2:**
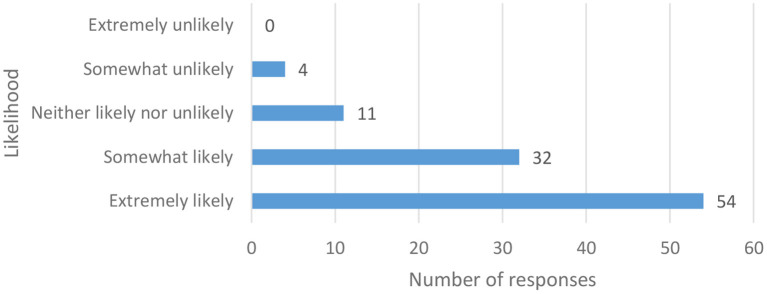
Likelihood of volunteering in sport events post-pandemic.

Thus, while most surveyed volunteers reported a clear intent to return to event volunteering post-pandemic, this research further revealed a worrying 15% considering themselves either “uncertain” or “unlikely to return” (see Figure A2), which represents a significant proportion of volunteer workforce. Additionally, of those volunteers “likely to return”, over 37% reported being only “somewhat” likely to return. Such an eventuality could have serious repercussions for the effective delivery of sport events. With a general trend in recent years of decline in formal volunteering (Cuskelly et al., 2006a; Department of Media Culture Sport, 2019), a further drop-off would exacerbate the situation and pose challenges to volunteer recruitment for sport events. Importantly, a significant proportion of volunteers who were unsure or unlikely to return to event volunteering were female (73.3%). While this figure would have been influenced by the fact that 59.4% of overall survey respondents were female, this remains an important finding. This is noteworthy because while females generally volunteer more than males (Downward et al., 2020), there is a higher proportion of males who volunteer in sport environments (Sport England, 2019)—a trend that some studies suggest also applies to sport events (Skirstad and Hanstad, 2013). If accurate, a further decline in female engagement would risk widening the gender gap all the more. However, the literature appears divided on this issue with some studies reporting higher levels of female engagement in sport event volunteering than males (Dickson and Benson, 2013; Nedvetskaya et al., 2015) while others suggest that sport events have an equal appeal to both males and females (Downward et al., 2005). Nonetheless, it can be argued that a decrease in female engagement in volunteering would pose a significant threat to volunteer recruitment programs for sport events.

Furthermore, most survey respondents uncertain about returning to volunteering were aged 45 or over, with the least confident group aged 55+. This finding should be of concern for managers in charge of volunteer programs, as 45+ demographics is traditionally the most likely group to volunteer (Downward et al., 2005). This also correlates with those people most worried about contracting the virus. The greatest proportion (68.4%) of surveyed respondents aged 55+ cited factors relating to fear of the virus as their primary concerns. By comparison, a much lower proportion (31.5%) of the younger age categories expressed similar views. This finding could be attributable to the identified increased risk posed by the virus to older generations (National Health Service, 2021). Similar trends were evident in the results of the volunteer interviews revealing a generally cautious mindset among older interviewees: “*Recruitment levels will fall because there is a cohort of volunteers who are of an older age range who will maybe just not feel safe, and maybe won't want to commit to [event volunteering] again*” (Volunteer B). This contrasted with higher levels of confidence expressed by younger volunteers: “*If [events] started to come back… I would go ahead and do it no problem at all*” (Volunteer D). In support, volunteer managers anticipated a shift in the volunteer age demographic. Less engagement was expected from older volunteers, while there was speculation that more younger people might engage due to reported eagerness for social interaction. As one volunteer manager expressed: “*The older age group of volunteers [would] fear coming back into those [event environments] and being exposed [to the virus]*” (Volunteer Manager C), while another stated: “*I actually think younger people have probably been impacted most during the pandemic… So, I think there's an opportunity to use events to try and bring some of those experiences back*” (Volunteer Manager F). Such a view finds support from a recent report by the Youth Sport Trust (2020) that found young people were hungry for increased social interaction due to the pandemic. Importantly, pre-existing patterns of increased youth volunteering contrasted by trends of decline among older generations appears to be a shift that has been accelerated during the COVID-19 pandemic (NFP Synergy, 2020). Arguably though, whether this trend would translate into increased event volunteering in the longer term remains to be seen. As one volunteer manager put it: “*I think [engagement] could go either way… We might see a shift with some people who didn't volunteer before now wanting to… [while] those who did volunteer might have decided actually, it's not for me [anymore]*” (Volunteer Manager C).

The critical revelation of this research is that sport event volunteering in the current circumstances is conditional on perceived levels of safety and changing environmental factors related to the stage and effectiveness of vaccination programs, infection rates, and COVID-19 Alert Levels, making it a rather complex issue. Although there was in varying degrees a general desire reported among volunteers interviewed to re-engage in event volunteering, it depended on the need to feel safe and robust safety measures being implemented by events. Volunteers expressed that their re-engagement would “*depend on the level of the pandemic*” (Volunteer G), be provisional on whether “*we are all vaccinated*” (Volunteer E), and conditional on whether appropriate “*safety procedures were put in place*” (Volunteer D). From the perspective of volunteer managers, concerns were surrounding potential new additional administrative burdens. These were related to the implementation of new COVID-19 related policies, such as risk assessments, supply of Personal Protective Equipment (PPE), social distancing and social isolation measures, sanitization, and track and trace procedures (Health Safety Executive, 2021; UK Government, 2021). However, these measures relate to guidance for organizations put in place or changed as things stand during the pandemic. It remains to be seen what the expectations will be post-pandemic. It can be reasonably expected that certain procedures will remain, at least for some time, as society re-emerges from the pandemic with a gradual return to pre-pandemic practices anticipated (He and Harris, 2020).

Historically, large sport events attract volunteers from local, national, and international spheres, with volunteer tourism becoming an increasing trend (Fairley et al., 2007; Smith and Holmes, 2009). This was reflected in the concerns of volunteer managers who cited travel restrictions and quarantine requirements as potential barriers to volunteer recruitment in the aftermath of the pandemic: “*International travel is going to be highly regulated, so I think for any events using international volunteers things could be very difficult*” (Volunteer Manager B). This suggests that a greater emphasis on the recruitment of volunteers from local areas is needed. However, the extent of this impact would be relative to national and international restrictions on travel, quarantine requirements, and the existing threat of the virus at the time. Even so, such an approach could bring benefits for sport events. Local recruitment could enhance the event legacy through the resulting increased localized skills transfer. In addition, recruitment from local areas would help generate a volunteer workforce possessing higher levels of local knowledge—often a weakness of transient events (Blackman et al., 2017).

A further consideration that emerged from the interview data is related to volunteer roles after the pandemic. While there was a general acceptance that traditional volunteer roles would remain, there was an expectation that some roles may require additional volunteers (e.g., relating to crowd management and signposting). This change might be needed to ensure compliance with safety measures such as segregation and limitations on the number of people in one place. Furthermore, potential new volunteer roles were also identified by volunteer managers. These included the proposal of new “COVID-19 volunteer” roles to support the implementation of COVID-19 safety procedures, such as the enforcement of social distancing, temperature checking, and symptom recording. Additionally, an increased need for volunteer roles relating to digital engagement was mentioned by volunteer managers: “*I think there will be a greater emphasis on digital engagement roles in the lead-up and during the event*” (Volunteer Manager F). These roles relate to pre-event support for recruitment and training, as well as during events as a means for enhancing online engagement among participants, fans, and spectators.

The identified potential changes to volunteer roles have implications for the need to increase human resource requirements in terms of the number of volunteers, their skill sets and, potentially, the number of volunteer managers. Reflective of the findings related to changes in volunteer demographics, sport events can expect heightened levels of engagement among the younger generations. An outcome of this nature may provide some further benefits for event organizers. Older people generally engage less with digital media than younger people (Green and Rossal, 2013; Matthews and Nazroo, 2015), whereas younger generations largely possess greater levels of digital literacy, which highlights a negative correlation between age and digital media competence (Andreou and Nicolaidou, 2019).

These findings combined with the potential increased need for digitally focused volunteer roles suggest that volunteer programs should be flexible in possibly targeting recruitment of younger demographics possessing the required digital skill sets. It can be argued that if volunteer managers do not adjust their recruitment approach but continue targeting the same demographics as pre-pandemic, they may risk experiencing a drop off in volunteer engagement. Furthermore, they may also struggle to recruit volunteers with the right skills and competencies required by the changing nature of certain volunteer roles. Establishing strategic links with Further and Higher Education providers was a suggestion that emerged from this study as an avenue to unlock access to potential skill-possessing volunteers. As noted by one interviewee: “*There will be technological implications for [volunteer programmes], [resource] implications, [and] human resource will probably be determined by skill set. I think it could be a question of, rather than going out to recruit [in a general way], being a bit more targeted in terms of how we approach recruiting volunteers*—*going to FE and HE organisations where there are relevant courses on digital engagement, social media, etc. to recruit these volunteers*” (Volunteer Manager F). Such an approach would have the potential to produce recognized mutual benefits for event organizers, academic institutions, and individual students alike (Ribarić et al., 2013).

Despite the challenges facing event organizers posed by the pandemic, some further positive outcomes emerged from the study. The subject of digital technology was a common theme not only related to the required skillsets for newly developed volunteer roles but also changes in volunteer management practices. The pandemic has seen a significant rise in the use of digital technology and social media across the population (Baker et al., 2020). Both volunteers and volunteer managers identified this change in behavior as an opportunity to make more effective use of technology in the organisation and delivery of volunteer programs. For example: “*We've never done online virtual briefings because we've never needed to. But actually, when we look at the events and what we could do better, we can improve the pre-event engagement to improve the experience… So, we will definitely be utilising the online platforms more moving forward*” (Volunteer Manager D). The main proposal was the use of blended online-offline approaches in the facilitation of volunteer orientation (e.g., delivery of online training, virtual tours of venues etc.). It was conceded that an element of in-person training would still be required for most roles. However, as changes in human interaction and learning have shifted during the pandemic (Vargo et al., 2021), it is evident that this is an avenue worth exploring. There are clear benefits to online training, including efficiencies around time, travel, and expense, in addition to added levels of convenience, flexibility, and practicalities such as recording training sessions and attendance monitoring (Mukhtar et al., 2020). Yet, it should be noted that such an approach may further contribute to the emerging digital divide between those with and without access (e.g., internet connection and computer equipment), confidence, and capability when it comes to engaging in online learning (Morgan, 2020; Vargo et al., 2021). Consideration should also be given to the additional resources needed in terms of people, time, technology, and finances to effectively implement such an approach (Morgan, 2020).

## Conclusions and Implications

This study aimed to critically examine the extent and reasons behind the theory-practice divide in volunteer–volunteer management relationship in the UK context of large sport events and investigate implications for volunteer programs within the context of the COVID-19 pandemic. The intention was to provide recommendations to inform event planning and delivery to enhance volunteer experiences and volunteer program outcomes.

This study identified an alarming deficiency in volunteer management resources throughout the sport event lifecycle, especially at the pre- and during event stages. A direct implication of what appears to be a theory-practice disconnect is in the resulting pressure on volunteer managers that may further restrict them in carrying out their roles as they are expected to, thus inhibiting their ability to effectively implement best practices. This reveals the discrepancy posited by this research between known good practice in theory (“how to do”) and the management behavior exhibited in the field (“how it's been done”). Such strain on volunteer managers would only be further exacerbated by the potential extra administrative workloads created by the need to implement additional COVID-19 related policies and procedures. A recommendation would, therefore, be for event organizers to consider greater support and resources allocation in terms of the staffing and funding of event volunteer programs to ensure sufficient capacity to implement known good practices in terms of volunteer management as well as address new challenges posed by the pandemic. Among such challenges are increased digitalization, the introduction of new volunteer roles to encourage compliance to COVID-19 procedures and help facilitate the safe operation of events upon the return of crowds. This would certainly lead to the associated increased need for volunteers that would be difficult to achieve considering the projected general reduction in the number of volunteers engaging or re-engaging in event volunteering post-pandemic and changing volunteer demographics.

Importantly, this study reported the uncertainty and nervousness among the volunteer workforce about re-engaging with sport events. Given the need for volunteer programs to overcome potential challenges in volunteer recruitment and retention post-pandemic, events must create positive volunteer expectations and experiences, which is closely linked to the resource element identified earlier. Evidence from this research suggested that effective volunteer management can positively influence the achievement of this aspiration; however, this requires sufficient resourcing to be done successfully (Cuskelly et al., 2006b; Allen and Bartle, 2013). Although this study did reveal a general desire and intention among volunteers to re-engage in event volunteering post-pandemic, the level of confidence appeared a determining factor. The decision to return to volunteering seemed to be conditional upon the effective rollout of a vaccination program, low levels of the virus in circulation, and the implementation of robust safety measures at events. Albeit not all these factors depend on volunteer managers along and is a joint effort of many stakeholders (e.g., various levels of government, international and national sport event governing bodies etc.), a further emphasis is placed on the need for effective volunteer management. The adoption of positive marketing approaches to recruitment by volunteer managers highlighting the benefits of volunteering is strongly advocated, with an emphasis on volunteering being the means to fulfill desires and experiences missed during the pandemic (e.g., social interaction, group identity, new experiences, use of existing or acquiring new skills, etc.). This practice combined with reassuring messages relating to the safety of events, and the maintenance of contact with existing volunteers during the pandemic are all considerations that could positively impact volunteer re-engagement. Additional considerations for enhancing the safety of volunteer programs and building confidence in volunteers may include the introduction of volunteer bubbles, outdoor volunteering opportunities, ventilation of indoor spaces, smaller volunteer teams, and flexible volunteer roles (e.g., remote working where possible).

This study further revealed a shift in the volunteer demographics, establishing a potential drop-off among the older generation and an increase in younger people willing to volunteer. These conclusions find support from recent reports identifying a decrease in active volunteerism among older people in the UK during the pandemic, and an increased desire to volunteer among the youth (NFP Synergy, 2020; Youth Sport Trust, 2020). A practical implication for sport events is, therefore, to consider the targeted recruitment of younger people, particularly through Further and Higher Education institutions, who have the skills and competencies that are most in-demand. This may help address the changes in the type of volunteer roles required by events in the aftermath of COVID-19. For example, opportunities for greater digital engagement between events and the public could be seized through digital volunteer roles—an area that could benefit from the engagement of digital-savvy younger volunteers. This study also identified the potential for volunteer programs to harness the increased digitalization of day-to-day life during the pandemic (Baker et al., 2020). This includes utilizing online training and virtual orientations to make volunteer engagement more convenient, while also enhancing learning (Mukhtar et al., 2020), improving volunteer experiences, and delivering organizational and personal efficiencies (e.g., decreasing venue hire costs or cutting on travel expenses). To achieve this, digital literacy becomes an essential factor that may attract new demographics yet exclude others from taking part based on either ability or access (or both).

Event organizations may be unable to influence national alert levels, vaccination programs, and global infection rates. Yet, as the sport event industry continues to re-emerge from the pandemic, the findings of this study suggest that the effective management of volunteer programs may play an even more important role in re-engaging previously active volunteers and attracting new ones than this was the case pre-pandemic.

### Research Limitations

The study took place at the height of the second wave of the COVID-19 pandemic in the UK (December 2020–February 2021). Albeit time-sensitive given the topic of research, this resulted in the study being impacted by the social restrictions in place at the time. Consequently, all data collection (survey and interviews) was conducted completely electronically. This would have limited participation to populations with access, confidence, and capability to engage in online-based research. Additionally, conducting the interviews remotely *via* a video link could potentially have inhibited the establishment of rapport and creation of an open environment. This could have influenced participant responses and prevented the interviewer from fully observing the environment and body language of the participant. These factors potentially could have limited the effective management of interviews and the authenticity of the data (Gratton and Jones, 2010).

Whilst able to provide critical data, the use of surveys is not always conducive for more complex questions and provide no opportunity to probe for further understanding (Jones, 2015). An ability to increase the amount of data captured *via* interviews instead of surveys could have produced more in-depth results. Furthermore, having an increased timescale for the study could have enabled broader data gathering and greater depth of analysis.

The study focused on volunteers and sport events based in Cardiff. The Cardiff-specific nature of the study may have limited the relevance of the research for other geographical locations and cultures. Yet, Cardiff is home to many national and international sport events which makes the findings of this study applicable beyond the Cardiff context. Additionally, the geographical focus and relatively small sample sizes for both the survey and interviews would have restricted the ability to generalize research findings to broader populations. Greater diversity in the population sample could have increased the potential of the study to gather more data and produce more broadly applicable results.

### Future Research

Intensified digitalization of volunteer engagement and volunteer management practices require further exploration by the events sector and academics alike. Future research should be endorsed to explore the engagement in event volunteering of previously non-volunteering populations, and the impact of the COVID-19 pandemic on event volunteering once the sport events industry re-opens its doors and associated volunteering opportunities. Newly developed government policies around QR codes and COVID passports to help combat the pandemic are of a particular interest, especially considering possible access restrictions. This leads to open questions regarding social stratification and key determinants of inclusion/exclusion from volunteer participation. Further consideration should also be given to the impact of the pandemic on the socio-psychosocial wellbeing of volunteers. Follow-up research with a greater number of participants conducted in different contexts and locations (either in the UK or another country) would allow for important comparisons that would further strengthen volunteer-related research, policy, and management practice to help the sector navigate its way beyond the current crisis.

## Data Availability Statement

The datasets presented in this article are not readily available because of Data Protection rules. Requests to access the datasets should be directed to gpower@cardiffmet.ac.uk.

## Ethics Statement

The studies involving human participants were reviewed and approved by Cardiff Metropolitan University Ethics Committee. The participants provided their written informed consent to participate in this study.

## Author Contributions

GP and ON were involved in the conceptualization and design of the study. GP recruited the participants and collected, processed, analyzed the data, and drafted the manuscript. ON critically revised the manuscript and approved the final submission. Both authors contributed to the article and approved the submitted version.

## Funding

This research was supported by Cardiff Metropolitan University.

## Conflict of Interest

The authors declare that the research was conducted in the absence of any commercial or financial relationships that could be construed as a potential conflict of interest.

## Publisher's Note

All claims expressed in this article are solely those of the authors and do not necessarily represent those of their affiliated organizations, or those of the publisher, the editors and the reviewers. Any product that may be evaluated in this article, or claim that may be made by its manufacturer, is not guaranteed or endorsed by the publisher.

## Appendix

**Table A1:** Volunteer demographics.

**Age group**	**Gender**			**Employment status**					
	**Male**	**Female**	**Non-disclosure**	**Employed (full-time)**	**Employed (part-time)**	**Retired**	**Student**	**Unemployed**	**Other**
18–25	2	9	0	4	1	0	6	0	0
26–35	5	7	0	7	2	0	1	1	0
36–45	1	4	0	4	0	0	1	0	1
46–55	8	15	0	18	2	0	0	0	3
56–65	16	13	1	10	5	12	0	1	2
65+	8	12	0	1	1	18	0	0	0
Overall	40	60	1	44	11	30	8	2	6

## References

[B1] Alexander A. Kim S. Kim D. (2015). Segmenting volunteers by motivation in the 2012 London Olympic Games. Tour. Manag. 47, 1–10 10.1016/j.tourman.2014.09.002

[B2] Allen J. Bartle M. (2013). Sport event volunteers' engagement: management matters. Manag. Leisure 19, 36–50. 10.1080/13606719.2013.849502

[B3] Allen J. B. Shaw S. (2009). “Everyone rolls up their sleeves and mucks in”: exploring volunteers' motivation and experiences of the motivational climate of a sporting event. Sport Manag. Rev. 12, 79–90. 10.1016/j.smr.2008.12.002

[B4] Andreou R. Nicolaidou I. (2019). Digital Literacy in Social Media: A Case Study. Kidmore End: Academic Conferences International Limited.

[B5] Baker C. Hutton G. Christie L. Wright S. (2020). COVID-19 and the Digital Divide. London: UK Parliament.

[B6] Bang H. Ross S. (2009). Volunteer motivation and satisfaction. J. Venue Event Manag. 1, 61–77.

[B7] Blackman D. Benson A. Dickson T. (2017). Enabling event volunteer legacies: a knowledge management perspective. Event Manag. 21, 233–251. 10.3727/152599517X14942648527473

[B8] Braun V. Clarke V. (2012). Thematic analysis, in APA Handbook of Research Methods in Psychology, Vol. 2, eds Cooper H. Camic P. Long D. Panter A. Rindskopf D. Sher K.. (Washington, DC: American Psychological Association) 57–71. https://sc.edu/study/colleges_schools/hrsm/research/publication_highlights/spte/journal_venue_and_event_management_archives/jvem_vol-1_iss-1/vol-1_iss-1_volunteer-motivation-satisfaction/

[B9] Catano V. Pond M. Kelloway E. (2001). Exploring commitment and leadership in volunteer organizations. Leader. Org. Dev. J. 22, 256–263. 10.1108/01437730110403187

[B10] Coleman R. (2002). Characteristics of volunteering in UK sport: Lessons from cricket. Manag. Leisure. 7, 220–238. 10.1080/1360671022000013710

[B11] Costa C. Chalip L. Green B. Simes C. (2006). Reconsidering the role of training in event volunteers' satisfaction. Sport Manag. Rev. 9, 165–182. 10.1016/S1441-3523(06)70024-9

[B12] Cox E. (2002). Rewarding volunteers: a study of participant responses t the assessment and accreditation of volunteer learning. Stud. Educ. Adults 34, 156–170. 10.1080/02660830.2002.11661468

[B13] Cuskelly G. Hoye R. Auld C. (2006a). Working with Volunteers in Sport. London: Routledge.

[B14] Cuskelly G. Taylor T. Hoye R. Darcy S. (2006b). Volunteer management practices and volunteer retention: a human resource management approach. Sport Manag. Rev. 9, 141–163. 10.1016/S1441-3523(06)70023-7

[B15] Department of Media Culture and Sport. (2019). Volunteers are One of the Most Valuable and Important Human Resources to the Amateur Sports. Available online at: https://www.civilsociety.co.uk/news/fewer-people-volunteering-says-dcms-survey.html (accessed March 11, 2020).

[B16] DeWalt K. DeWalt B. (2011). Participant Observation: A Guide for Fieldworkers, 2nd Edn. Plymouth: AltaMira Press.

[B17] Dickson T. Benson A. (2013). London 2012 games makers: Towards redefining legacy, in Policy: Creating a lasting legacy from the 2012 Olympic and Paralympic Games. (London: Department for Culture Media and Sport) 1–7.

[B18] Doherty A. (2009). The volunteer legacy of a major sport event. J. Policy Res. Tour. Leisure Events 1, 185–207. 10.1080/19407960903204356

[B19] Downward P. Hallmann K Rasciute S. (2020). Volunteering and leisure activity in the united kingdom: a longitudinal analysis of males and females. Nonprofit Volunt. Sector Q. 49, 757–775. 10.1177/0899764020901815

[B20] Downward P. Lumsdon L. Ralston R. (2005). Gender differences in sports event volunteering: insights from Crew 2002 at the XVII Commonwealth Games. Manag. Leisure 10, 219–236. 10.1080/13606710500348086

[B21] Dunn J. Chambers S. Hyde M. (2016). Systematic review of motives for episodic volunteering. Voluntas 27, 425–464. 10.1007/s11266-015-9548-425248403

[B22] Dwyer M. Pasini M. De Dominicis S. Righi E. (2020). Physical activity: benefits and challenges during the COVID-19 pandemic. Scand. J. Med. Sci. Sports 30, 1291–1294. 10.1111/sms.13710 32542719 PMC7323175

[B23] Fairley S. Kellett P. Green B. (2007). Volunteering abroad: motives for travel to volunteer at the Athens Olympic Games. J. Sport Manag. 21, 41–58. 10.1123/jsm.21.1.41

[B24] Farmer S. Fedor D. (1999). Volunteer participation and withdrawal. Nonprofit Manag. Leader. 9, 349–367. 10.1002/nml.9402

[B25] Finkelstein M. (2008). Volunteer satisfaction and volunteer action: a functional approach. Soc. Behav. Pers. Int. J. 36, 9–18. 10.2224/sbp.2008.36.1.9

[B26] Gallarza M. Arteaga F. Gil-Saura I. (2013). The value of volunteering in special events: a longitudinal study. Ann. Tourism Res. 40, 105–131. 10.1016/j.annals.2012.08.001

[B27] Gratton C. Jones I. (2010). Research Methods for Sport Studies. 2nd Edn. New York, NY: Routledge.

[B28] Green M. Rossal P. (2013). Digital Inclusion Evidence Review. London: Age UK.

[B29] Hallmann K. Downward P. Dickson G. (2018). Factors influencing time allocation of sport event volunteers. Int. J. Event Fest. Manag. 9, 316–331. 10.1108/IJEFM-01-2018-0004

[B30] Hallmann K. Harms G. (2012). Determinants of volunteer motivation and their impact on future voluntary engagement. Int. J. Event Fest. Manag. 3, 272–281. 10.1108/17582951211262701

[B31] He H. Harris L. (2020). The impact of Covid-19 pandemic on corporate social responsibility and marketing philosophy. J. Bus. Res. 116, 176–182. 10.1016/j.jbusres.2020.05.030 32457556 PMC7241379

[B32] Health and Safety Executive (2021). Making Your Workplace COVID-Secure During the Coronavirus Pandemic. Available online at: https://www.hse.gov.uk/coronavirus/working-safely/index.htm (accessed March 2, 2021).

[B33] Hiltrop J. (1999). The quest for the best: human resource practices to attract and retain talent. Eur. Manag. J. 17, 422–430. 10.1016/S0263-2373(99)00022-5

[B34] Hoye R. Smith A. Nicholson M. Stewart B. (2018). Sport Management: Principles and Applications. 5th Edn. London: Routledge.

[B35] Jones I. (2015). Research Methods for Sport Studies. 3rd Edn. London: Routledge.

[B36] Khoo S. Engelhorn R. (2011). Volunteer motivations at a National Special Olympics event. Adapt. Phys. Activity Q. 28, 27–39. 10.1123/apaq.28.1.27 21282846

[B37] Kim E. Fredline L. Cuskelly G. (2018). Heterogeneity of sport event volunteer motivations: a segmentation approach. Tour. Manag. 68, 375–386. 10.1016/j.tourman.2018.04.004

[B38] Kirstiansen E. Skirstad B. Parent M. (2015). ‘We can do it’: community, resistance, social solidarity, and long-term volunteering at a sport event. Sport Manag. Rev. 18, 256–267. 10.1016/j.smr.2014.06.002

[B39] Lee Y. Kim M. Koo J. (2016). Lee, The impact of social interaction and team member exchange on sport event volunteer management. Sport Manag. Rev. 19, 550–562. 10.1016/j.smr.2016.04.005

[B40] Matthews K. Nazroo J. (2015). Understanding Digital Engagement in Later Life. London: UK Government.

[B41] Miles L. Shipway R. (2020). Exploring the covid-19 pandemic as a catalyst for stimulating future research agendas for managing crises and disasters at international sport events. Event Manag. 24, 537–552. 10.3727/152599519X15506259856688

[B42] Millette V. Gagné M. (2008). Designing volunteers' tasks to maximise motivation, satisfaction and performance: the impact of job characteristics on volunteer engagement. Motiv. Emotion 32, 11–22. 10.1007/s11031-007-9079-4

[B43] MIND. (2017). https://www.mind.org.uk/information-support/t Mental Health Problems Introduction: Stigma Misconceptions. Available online at: https://www.mind.org.uk/information-support/types-of-mental-health-problems/mental-health-problems-introduction/stigma-misconceptions/?gclid=EAIaIQobChMIr6Kb64eN7wIVXoBQBh2iWwACEAAYAiAAEgLRq_D_BwE (accessed February28, 2021).

[B44] Morgan H. (2020). Best practices for implementing remote learning during a pandemic. Clear. House J. Educ. Strat. Issues Ideas 93, 135–141. 10.1080/00098655.2020.175148035126224

[B45] Mukhtar K. Javed K. Arooj M. Sethi A. (2020). Mukhtar, K., advantages, limitations and Recommendations for online learning during COVID-19 pandemic era. Pak. J. Med. Sci. 36, 27–31. 10.12669/pjms.36.COVID19-S4.2785 32582310 PMC7306967

[B46] Musick M. Wilson J. (2008). Volunteers. A Social Profile. Bloomington: Indiana University Press.

[B47] National Health Service (2021). Covid-19: People at Higher Risk. Available online at: https://www.nhs.uk/conditions/coronavirus-covid-19/people-at-higher-risk/ (accessed February 28, 2021).

[B48] Nedvetskaya O. Girginov V. (2017). Volunteering legacy of the London 2012 Olympics, in Legacies and Mega-Events: Fact or Fairy Tales?, eds Brittain I. Bocarro J. Swart K. Byers T. (London: Routledge) 61–78.

[B49] Nedvetskaya O. Purcell R. Hastings A. (2015). Looking back at London 2012: recruitment, selection and training of games makers, in The London Olympics and Urban Development: The Mega-Event City, eds Poynter G. Viehoff V. Li Y (London: Routledge), 293–306.

[B50] NFP Synergy (2020). Are Young People Replacing Older People as the Key Volunteering Group? London: NFP Synergy.

[B51] Nichols G. (2014). Volunteers in Sport. London: Routledge.

[B52] Nichols G. Hogg E. Knight C. Storr R. (2019). Selling volunteering or developing volunteers? Approaches to promoting sports volunteering. Volunt. Sect. Rev. 10, 3–18. 10.1332/204080519X1547820012513223599727

[B53] Office of National Statistics (2017). Changes in the Value and Division of Unpaid Volunteering in the UK: 2000 to 2015. Available online at: https://www.ons.gov.uk/economy/nationalaccounts/satelliteaccounts/articles/changesinthevalueanddivisionofunpaidcareworkintheuk/2015 (accessed March 11, 2020).

[B54] Olympic Games (2012). The Benefits of Hosting London 2012 will be Ongoing. Available online at: https://www.olympic.org/news/benefits-of-hosting-london-2012-will-be-ongoing (accessed January 4, 2020).

[B55] Phillips L. Phillips M. (2010). Volunteer motivation and reward preference: An empirical study of volunteerism in a large, not-for-profit organisation. SAM Adv. Manag. J. 75, 12–22. https://www.researchgate.net/profile/Radha-Iyer-3/post/Students-volunteering/attachment/59d64f7979197b80779a89ba/AS%3A497876854939648%401495714500065/download/Volunteer+Motivation+and+Reward.pdf

[B56] Ribarić H. Nimac K. Nad M. (2013). Volunteering and competitiveness on the labour market in times of crisis: students' attitudes. Tour. South. East. Eur. 2, 217–230. https://www.researchgate.net/deref/https%3A%2F%2Fsearch.proquest.com%2Fopenview%2Fc55a3b44596f57574f675e8e33dcaacf%2F1%3Fpq-origsite%3Dgscholar%26cbl%3D1936348

[B57] Roza L. Shachar I. Meijs L. Hustinx L. (2017). The nonprofit case for corporate volunteering: a multi-level perspective. Serv. Indust. J. 37, 746–765. 10.1080/02642069.2017.1347158

[B58] Schlesinger T. Nagel S. (2018). Individual and contextual determinants of stable volunteering in sport clubs. Int. Rev. Sociol. Sport 53, 101–121. 10.1177/1012690216638544

[B59] Shahidi S. Stewart-Williams J. Hassani F. (2020). Physical activity during COVID-19 quarantine. Acta Paediatr. 109, 2147–2148. 10.1111/apa.15420 32557827 PMC7323361

[B60] Sheptak R. Menaker B. (2020). When sport event work stopped: exposure of sport event labor precarity by the COVID-19 pandemic. Int. J. Sport Commun. 13, 427–435. 10.1123/ijsc.2020-0229

[B61] Shin S. Kleiner B. (2003). How to manage unpaid volunteers in organizations. Manag. Res. News 26, 63–71. 10.1108/01409170310784005

[B62] Skinner J. Edwards A. Corbett B. (2015). Research Methods for Sport Management. London: Routledge.

[B63] Skirstad B. Hanstad D. (2013). Gender matters in sport event volunteering. Manag. Leisure 18, 316–331. 10.1080/13606719.2013.809188

[B64] Smith A. (2020). The BASES expert statement on physical activity and exercise during covid-19 “lockdowns” and “restrictions”. Sport Exerc. Sci. 65, 13–16. https://www.bases.org.uk/imgs/bases_expert_statement298.pdf

[B65] Smith K. Holmes K. (2009). Researching volunteers in tourism: going beyond. Ann. Leisure Res. 12, 403–420. 10.1080/11745398.2009.9686831

[B66] Sport England (2019). Active Lives Adult Survey. London: Sport England.

[B67] Sport and Recreation Alliance (2018). Sports Club Survey Report 2017/18. London: Sport and Recreation Alliance.

[B68] Studer S. von Shnurbein G. (2013). Organizational factors affecting volunteers: a literature review on volunteer coordination. Voluntas 24, 403–440. 10.1007/s11266-012-9268-y

[B69] Surujlal J. Dhurup M. (2008). ‘Volunteers’ perceptions of benefits derived from volunteering: an empirical study'. South Afr. J. Res. Sport Phys. Educ. Recreat. 30, 105–116. 10.4314/sajrs.v30i1.25987

[B70] Taylor T. Darcy S. Hoye R. Cuskelly G. (2006). Using psychological contract theory to explore issues in effective volunteer management. Eur. Sport Manag. Q. 6, 123–147. 10.1080/16184740600954122

[B71] Taylor T. Doherty A. McGraw P. (2008). Managing People in Sport Organizations: A Strategic Human Resource Management Perspective. Oxford: Butterworth-Heinmann.

[B72] Tokyo (2020). Olympic Games Postponed to 2021. Available online at: https://tokyo2020.org/en/news/joint-statement-from-international-olympic-committee-and-tokyo2020 (accessed January 4, 2020).

[B73] Torales J. O'Higgins M. Castaldelli-Maia J. Ventriglio A. (2020). The outbreak of COVID-19 coronavirus and its impact on global mental health. Int. J. Soc. Psychiatry 66, 317–320. 10.1177/0020764020915212 32233719

[B74] UK Government (2019). Wellbeing in Mental Health: Applying all our Health. Available online at: https://www.gov.uk/government/publications/wellbeing-in-mental-health-applying-all-our-health/wellbeing-in-mental-health-applying-all-our-health (accessed February 28, 2021).

[B75] UK Government (2021). Working Safely During Coronavirus (Covid-19). Available online at: https://www.gov.uk/guidance/working-safely-during-coronavirus-covid-19 (accessed March 2, 2021).

[B76] Vargo D. Zhu L. Benwell B. Yan Z. (2021). Digital technology use during COVID-19 pandemic: a rapid review. Hum. Behav. Emerg. Technol. 3, 13–24. 10.1002/hbe2.242

[B77] Wackerhage H. Everett R. Krüger K. Murgia M. Simon P. Gehlert S.. (2020). Sport and exercise and COVID-19, the disease caused by the SARS-CoV-2 coronavirus. German J. Sports Med. 71, 1–12. 10.5960/dzsm.2020.441

[B78] Yeung A. (2004). The octagon model of volunteer motivation: results of a phenomenological analysis. Voluntas Int. J. Volunt. Nonprofit. 15, 21–46. 10.1023/B:VOLU.0000023632.89728.ff

[B79] Youth Sport Trust (2020). Evidence Paper: The Impact of Covid-19 Restrictions on Children and Young People, Loughborough: Youth Sport Trust.

